# Investigation of Tau as a self‐antigen that drives T cell immunity in a tauopathy mouse model

**DOI:** 10.1002/alz.086167

**Published:** 2025-01-03

**Authors:** Hao Hu, Jason D Ulrich, David M. Holtzman

**Affiliations:** ^1^ Department of Neurology, Washington University School of Medicine, St. Louis, MO USA; ^2^ Knight Alzheimer Disease Research Center, St. Louis, MO USA; ^3^ Hope Center for Neurological Disorders, Washington University School of Medicine, St. Louis, MO USA; ^4^ Washington University School of Medicine in St Louis, St. Louis, MO USA

## Abstract

**Background:**

Alzheimer’s disease (AD) is characterized by the aggregation and accumulation of proteins including amyloid‐β and tau. We previously compared the immunological milieus in the brain of mice with amyloid deposition or tau aggregation and found that mice with tauopathy but not amyloid developed a unique adaptive immune response with markedly increased activated T cells in areas with tau pathology. T cell depletion blocked tau‐mediated neurodegeneration. These indicate a pathogenic role for T cells in tau‐mediated neurodegeneration. Which antigens are presented to and activate T cells during tauopathy is unknown. Individuals developing AD pathology displayed increased tau species including with microtubule binding region (MTBR) in the extracellular space of CNS, which is rare in healthy individuals. These regions can be potentially recognized by T cells and lead to their activation. Also, neoepitopes can be generated from tau caused by the formation of neurofibrillary tangles, which contain tau hyperphosphorylation, acetylation, citrullination, ubiquitination. These may trigger an “allogeneic” T cell response. Thus, we hypothesize that tau could be a potential antigen that drives an autoimmune‐like response during development of tau pathology in a tauopathy mouse model.

**Method:**

We intend to make use of an online epitope prediction tool ([https://www.iedb.org/]) to select candidate peptides derived from tau and test their immunological response by immunizing the mice with synthetic peptides. We will also utilize an unbiased TCR (T cell receptor) enrichment analysis to generate antigen (tau)‐specific T cells and test their pathological role during tau pathology.

**Result:**

In our preliminary experiments, we successfully predicted multiple antigenic epitopes to MHC‐I or MHC‐II derived from full‐length human P301S tau mutant protein. We found several tau peptides from the top‐ranking predicted sequences can elicit a robust T‐cell response in P301S mice. We also generated T‐cell hybridomas that specifically recognize tau:225‐240 peptides.

**Conclusion:**

Tau‐specific T cells can develop in P301S mice. Completion of this project will attempt to provide a detailed immunological mechanism of how T cells become activated during tau pathology in AD. It will also provide new approaches to target these T cells or engineer therapeutic T cells to develop methods to treat tauopathies including AD.

